# Supplementary material for: Effects of foliar fungicide on yield, micronutrients, and cadmium in grains from historical and modern hard winter wheat genotypes
Source: PLoS One. 2021 Mar 4;16(3):e0247809. doi: 10.1371/journal.pone.0247809 (PMC7932086; doi:10.1371/journal.pone.0247809)
Supplement: S1 Fig — (DOCX) [file pone.0247809.s001.docx]

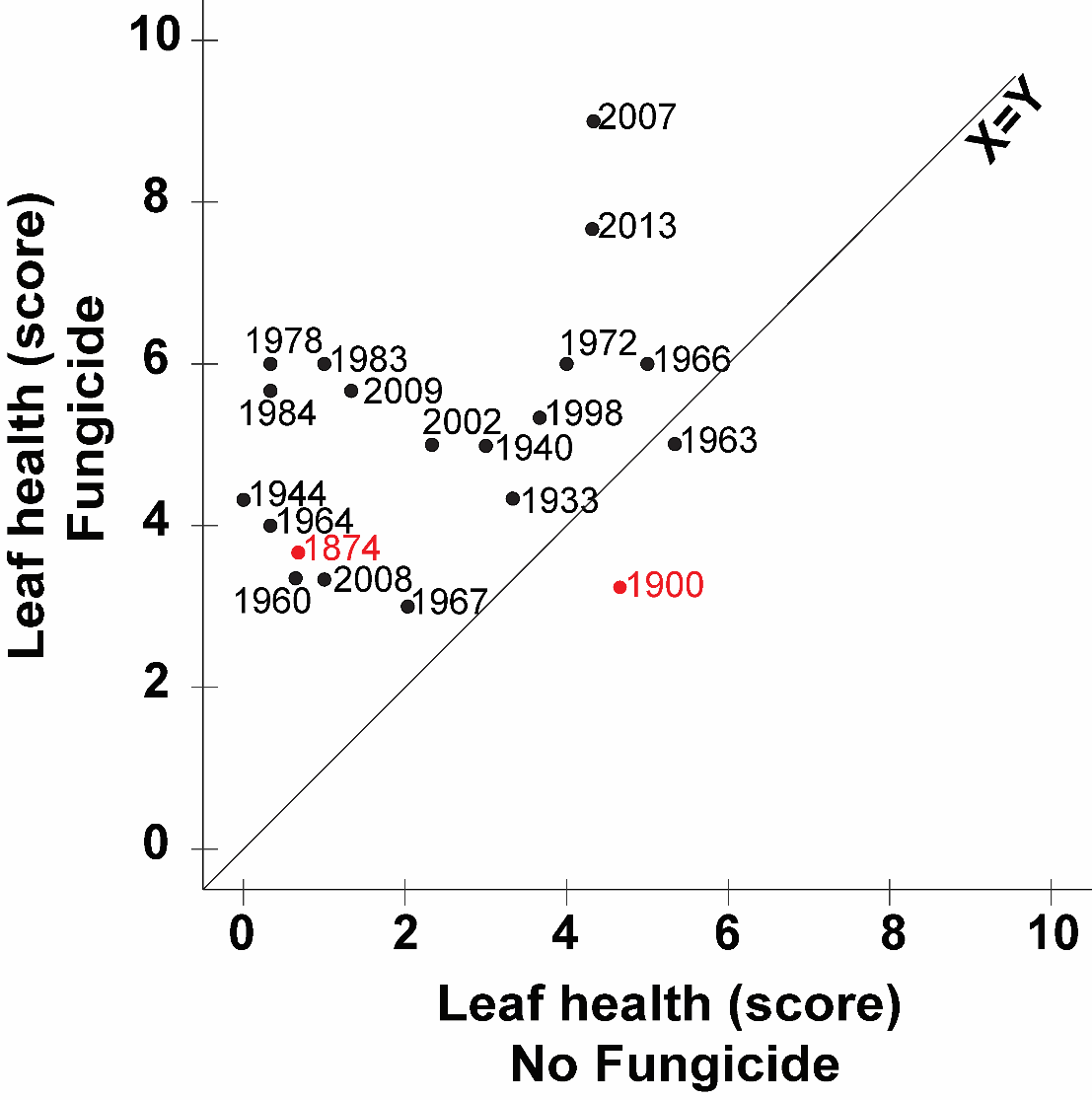

**S1 Fig.** Scatter plot comparison of the leaf health score in the presence and absence of fungicide from 2017 (2018 data not available).
