# Supplementary material for: Effects of foliar fungicide on yield, micronutrients, and cadmium in grains from historical and modern hard winter wheat genotypes
Source: PLoS One. 2021 Mar 4;16(3):e0247809. doi: 10.1371/journal.pone.0247809 (PMC7932086; doi:10.1371/journal.pone.0247809)
Supplement: S2 Fig — Linear regressions of A) grain diameter and B) thousand kernels weight (TKW) by registration year for a historic set of 18 wheat cultivars released between 1933 and 2013, which were grown in a split-plot field experiment in the presence or absence of fungicide application at the Eastern Nebraska Research and Education Center in 2017 and 2018. The regression lines were adjusted for growing year effects and the shaded area around each regression line represents the 95% confidence interval. The landraces ‘Turkey’ (1874) and ‘Kharkof’ (1900) were not included in the regression analysis. (DOCX) [file pone.0247809.s002.docx]

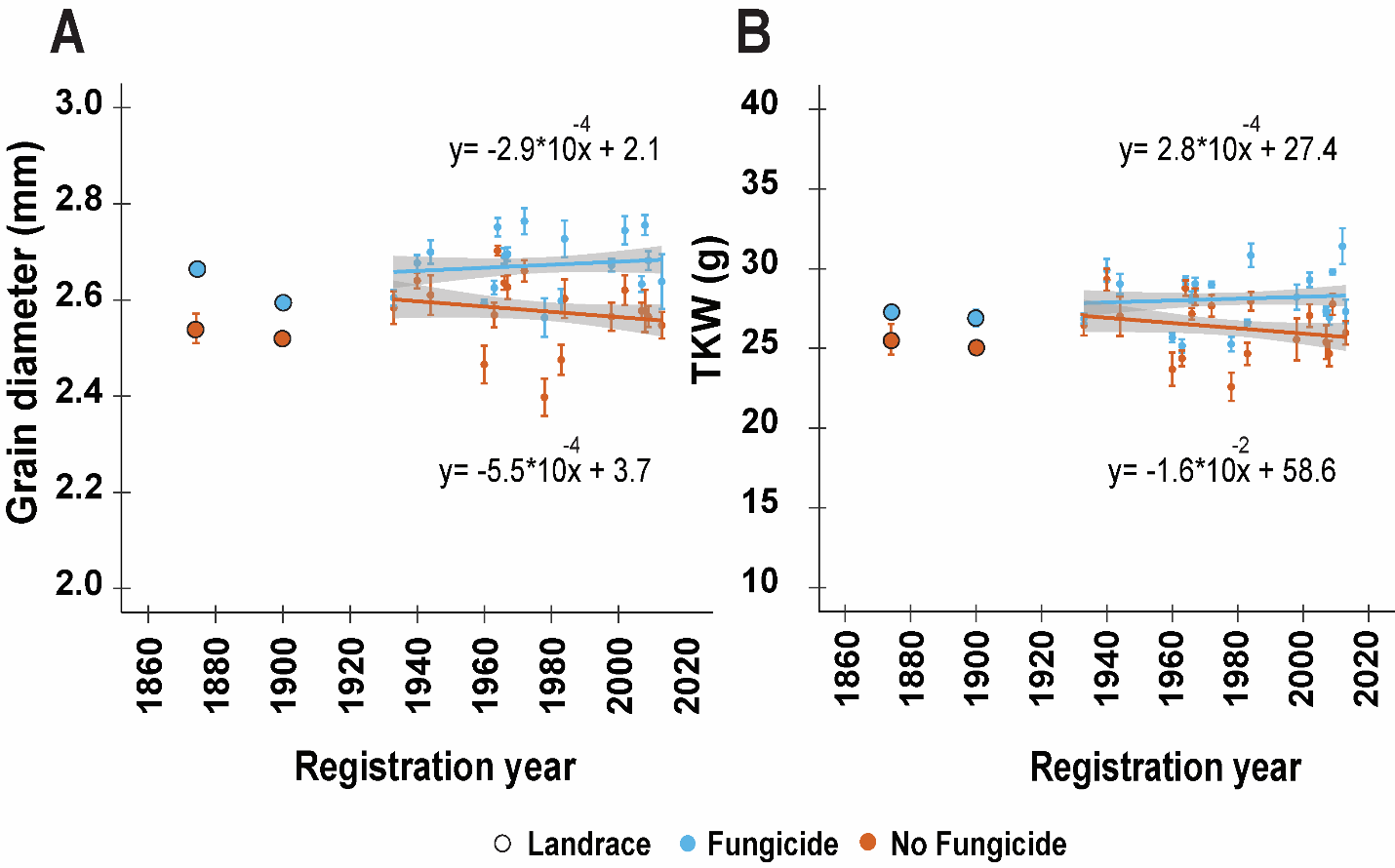

**S2 Fig.** Linear regressions of A) grain diameter and B) thousand kernels weight (TKW) by registration year for a historic set of 18 wheat cultivars released between 1933 and 2013, which were grown in a split-plot field experiment in the presence or absence of fungicide application at the Eastern Nebraska Research and Education Center in 2017 and 2018. The regression lines were adjusted for growing year effects and the shaded area around each regression line represents the 95% confidence interval. The landraces ‘Turkey’ (1874) and ‘Kharkof’ (1900) were not included in the regression analysis.
