# Supplementary material for: Effects of foliar fungicide on yield, micronutrients, and cadmium in grains from historical and modern hard winter wheat genotypes
Source: PLoS One. 2021 Mar 4;16(3):e0247809. doi: 10.1371/journal.pone.0247809 (PMC7932086; doi:10.1371/journal.pone.0247809)
Supplement: S1 Table — (DOCX) [file pone.0247809.s003.docx]

**S1 Table.** Pearson correlation coefficients between the economic traits, grain dimensions, phytate, micronutrients, and cadmium of wheat grains across environments (2017-2018) in the presence and absence of fungicide.

| **Variable** | **Fungicide** | **No Fungicide** |
| --- | --- | --- |
| **Yield** | 0.62** | 0.62** |
| **Protein** | 0.49* | 078*** |
| **Phytate** | 0.04 | 0.52* |
| **Mg** | -0.05 | 0.11 |
| **P** | 0.23 | 0.09 |
| **Mn** | 0.63** | 0.52* |
| **Fe** | 0.4 | 0.62** |
| **Zn** | 0.37 | 0.61** |
| **Cd** | 0.72*** | 0.61*** |
| **Diameter** | 0.46* | 0.65** |
| **TKW** | 0.64** | 0.64** |

*Significant at the 0.05 probability level.
**Sig Significant at the 0.01 probability level.
***Significant at the 0.001 probability level.
