# Supplementary material for: Effects of foliar fungicide on yield, micronutrients, and cadmium in grains from historical and modern hard winter wheat genotypes
Source: PLoS One. 2021 Mar 4;16(3):e0247809. doi: 10.1371/journal.pone.0247809 (PMC7932086; doi:10.1371/journal.pone.0247809)
Supplement: Attachment — Submitted filename: Response to reviewer R2.docx [file pone.0247809.s006.docx]

**Response to reviewer comments on PONE-D-20-20368R1**

Authors’ responses are in blue

**Reviewer 3**

Q: Thank you for this very interesting study about macronutrients and Cadmium contamination of seeds. Cd can be added to the soil through natural and anthropogenic activities, fertilizers derived from phosphate rock. Authors conclude a relationship between biotic stress and the increased Cd content in the absence of fungicide treatment. Suggestions and remarks of two reviewers have been considered already, however, I suggest some minor changes.
Biotic stresses could arise by many reasons. If data to the resistance level against fungal diseases in the non fungicide treated control are available, please provide and discuss the relationship of infection to the Cd content. In lines 288 to 293 you mentioned, that "a rainy June allowed for higher disease pressure after flowering with leaf rust (Puccina triticina), bacterial leaf streak (Xanthomonas translucens), and fusarium head blight (Fusarium graminearum) as the main diseases observed". Please include some information about the resistance level of the cultivars, especially regarding yellow rust and leaf rust. Fungal (leaf) pathogens are strong sinks for nutrients, and probably for heavy metals.
A: Thank you for these comments. As foliar fungal pathogens compete for nutrients with plants, the level of infection can affect the partitioning of carbohydrates, nitrogen, and micronutrients in the grains. Therefore, we agree that addressing the relationship between genotype susceptibility to foliar fungal diseases and grain micronutrients and Cd will strengthen this manuscript. In this revision, we report the genotype susceptibility to leaf and stripe rust in **Table 1** as susceptible (S), moderately susceptible (MS), and moderately resistant (MR). To investigate the relationship between genotype susceptibility to foliar diseases and mineral elements in grains, the cultivars were divided into two groups: 1) S and 2) MS/MR. Then, the difference in grain mineral elements between fungicide treatments was compared between these two groups. No significant differences were found for Cd or any micronutrient (*P* > 0.05). However, since most of the cultivars included in this study were susceptible to foliar diseases, the size of the MS/MR group was very small (N=6 for leaf rust and N=3 for stripe rust). Therefore, although no evident relationship between genotype susceptibility to foliar diseases and mineral elements in grains was found in this study, additional research with a balanced experimental design would be more conclusive. This is now included in the Results and Discussion sections [**lines 418-422, 579-582 (track changes);** **lines 412-416, 556-559 (clean)**].

Q: Furthermore, fungicide treatment could not be generalized. In case of Metconazole as active compound of Caramba, a typical single-site fungicide has been used. It should be discussed, that other compounds, e.g. strobilurins, could result in contradictive results due to a "greening effect".

A: In this study, the application of fungicide (metconazole) efficiently reduced disease pressure, resulting in increased yield and GPC while simultaneously diluting the mineral elements in the grains when compared with untreated plants. However, this effect of fungicide should not be generalized as other active compounds could yield different results. For instance, fungicides based on strobilurins and carboxamides, besides controlling diseases, impart physiological effects such as delaying senescence. Thus, other fungicides could allow for a greater deposition of carbohydrates or micronutrients in grains and yield different results from those presented here. This discussion point has been added to the Discussion [**lines 566-575 (track changes); lines 546-555 (clean)**].
